# Sex differences in lipid metabolism are affected by presence of the gut microbiota

**DOI:** 10.1038/s41598-018-31695-w

**Published:** 2018-09-07

**Authors:** Annemarie Baars, Annemarie Oosting, Mirjam Lohuis, Martijn Koehorst, Sahar El Aidy, Floor Hugenholtz, Hauke Smidt, Mona Mischke, Mark V. Boekschoten, Henkjan J. Verkade, Johan Garssen, Eline M. van der Beek, Jan Knol, Paul de Vos, Jeroen van Bergenhenegouwen, Floris Fransen

**Affiliations:** 1 Danone Nutricia Research, Utrecht, The Netherlands; 2 Department of Pediatrics, University of Groningen, University Medical Centre Groningen, Groningen, The Netherlands; 3 Microbial Physiology, Groningen Biomolecular Sciences and Biotechnology Institute, University of Groningen, Groningen, The Netherlands; 4 Top Institute Food and Nutrition, Wageningen, The Netherlands; 5 Laboratory of Microbiology, Wageningen University, Wageningen, The Netherlands; 6 Nutrition, Metabolism & Genomics Group, Division of Human Nutrition, Wageningen University, Wageningen, The Netherlands; 7 Faculty of Science, Utrecht Institute for Pharmaceutical Sciences, Utrecht University, Utrecht, The Netherlands; 8 Department Pathology and Medical biology, section Immunoendocrinology, University of Groningen, University Medical Centre Groningen, Groningen, The Netherlands

## Abstract

Physiological processes are differentially regulated between men and women. Sex and gut microbiota have each been demonstrated to regulate host metabolism, but it is unclear whether both factors are interdependent. Here, we determined to what extent sex-specific differences in lipid metabolism are modulated via the gut microbiota. While male and female Conv mice showed predominantly differential expression in gene sets related to lipid metabolism, GF mice showed differences in gene sets linked to gut health and inflammatory responses. This suggests that presence of the gut microbiota is important in sex-specific regulation of lipid metabolism. Further, we explored the role of bile acids as mediators in the cross-talk between the microbiome and host lipid metabolism. Females showed higher total and primary serum bile acids levels, independent of presence of microbiota. However, in presence of microbiota we observed higher secondary serum bile acid levels in females compared to males. Analysis of microbiota composition displayed sex-specific differences in Conv mice. Therefore, our data suggests that bile acids possibly play a role in the crosstalk between the microbiome and sex-specific regulation of lipid metabolism. In conclusion, our data shows that presence of the gut microbiota contributes to sex differences in lipid metabolism.

## Introduction

Accumulating evidence indicates that metabolic homeostasis is differentially regulated between men and women^1–4^. Particularly in humans and certain animals, sex-specific effects in lipid and cholesterol metabolism are well-known and associated with different risk profiles for cardiovascular disease^5–7^. Mechanistic studies in mice showed marked male-female gene expression differences in the offspring of dams that received a western style diet^6^. Moreover, sex-specific differences were already observed at weaning, with changes in physiological parameters such as bodyweight and plasma lipids. In addition, whereas the males showed differences in genes related to developmental functions, the female offspring showed primarily differences in lipid metabolism genes, including hepatic cholesterol synthesis. These sex-specific developmental effects have been related to differences in circulating sex hormones which affect the metabolic system^8–13^.

Numerous studies have shown the importance of the gut microbiota for the metabolism of the host (reviewed by^14^). Velagapudi and colleagues described that the gut microbiota affected energy metabolism and host lipid metabolism, leading to decreased triglyceride levels in serum but increased levels in adipose and liver tissue in conventional (Conv) mice compared to germ-free (GF) mice^15^. One possible mechanism by which the gut microbiota might influence metabolic homeostasis is via changing the composition and quantity of bile acids. Several species of bacteria indeed can express bile acid transforming enzymes. Bile acids are increasingly recognized as mediators in the crosstalk between the microbiome and host lipid and cholesterol-related metabolic gene sets^16^.

Bile acids are synthesized from cholesterol in the liver. Subsequently, bile acids are conjugated to either taurine (predominant in mice) or glycine (predominant in humans) and secreted via bile into the gastrointestinal tract. Bile acids are primarily needed for generating bile flow and for solubilization of dietary lipids, but are also regarded as signaling molecules through activation of bile acid receptors such as farnesoid X receptor (Fxr) and Tgr5 (reviewed by^16–18^). Host bile acid production is tightly controlled through a feedback loop^19,20^. Here, bile acids activate ileal Fxr leading to the release of fibroblast growth factor 15 (Fgf15; known as FGF19 in humans). Increased plasma levels of Fgf15 subsequently inhibit liver bile acid synthesis through inhibition of the enzyme cholesterol 7-alpha-hydroxylase (Cyp7a1).

Sayin and colleagues (2013) proposed that gut microbiota has major effects on bile acid homeostasis through modulation of Fxr signaling in the gut^21,22^. Data from experiments with GF mice indicated that both bile acid levels and composition are regulated by the intestinal microbiota. The gut microbiota metabolizes bile acids through deconjugation and 7α/β-dehydroxylation to increase their hydrophobicity^23^. A broad spectrum of intestinal anaerobic bacteria, such as various members of the phyla Firmicutes and Bacteroidetes, express the bile salt hydrolase (BSH) gene which is necessary to deconjugate bile acids for further processing into secondary bile acid species through 7α/β-dehydroxylation^16,23,24^. However, only a limited number of intestinal microbes, such as Bacteroides, Clostridia, *E. coli* and Ruminococcus, express the enzymes needed for 7α/β-dehydroxylation. Moreover, data on specific bacterial bile acid modifications showed profound effects on intestinal and hepatic lipid-related pathways, illustrating the importance of bile acid transformation for host metabolism^25^.

Over the years, it has become clear that gut microbiota composition is, at least in part, sex-dependent^26–28^. In contrast, potential sex differences in bile acid homeostasis remain mostly unexplored. Only a few studies demonstrated sex differences in bile acid concentrations and composition in mice^29–31^. Several studies have indeed shown that sex and gut microbiota each regulate host metabolism, but it is unclear whether both factors are interdependent^5–7,15,32,33^. In the present study, we determined to what extent sex-specific differences in lipid metabolism are modulated via the gut microbiota. Further, we explored the role of bile acids as mediators in the cross-talk between the host lipid metabolism and the microbiome. To this end, ileal gene expression was measured by transcriptome-wide microarray analysis in GF and Conv adult male and female mice. In addition, we determined sex differences in serum bile acids of GF and Conv mice and microbiota composition in Conv animals.

## Results

### Presence of gut microbiota contributes to sex differences in lipid metabolism

**Figure 1:**
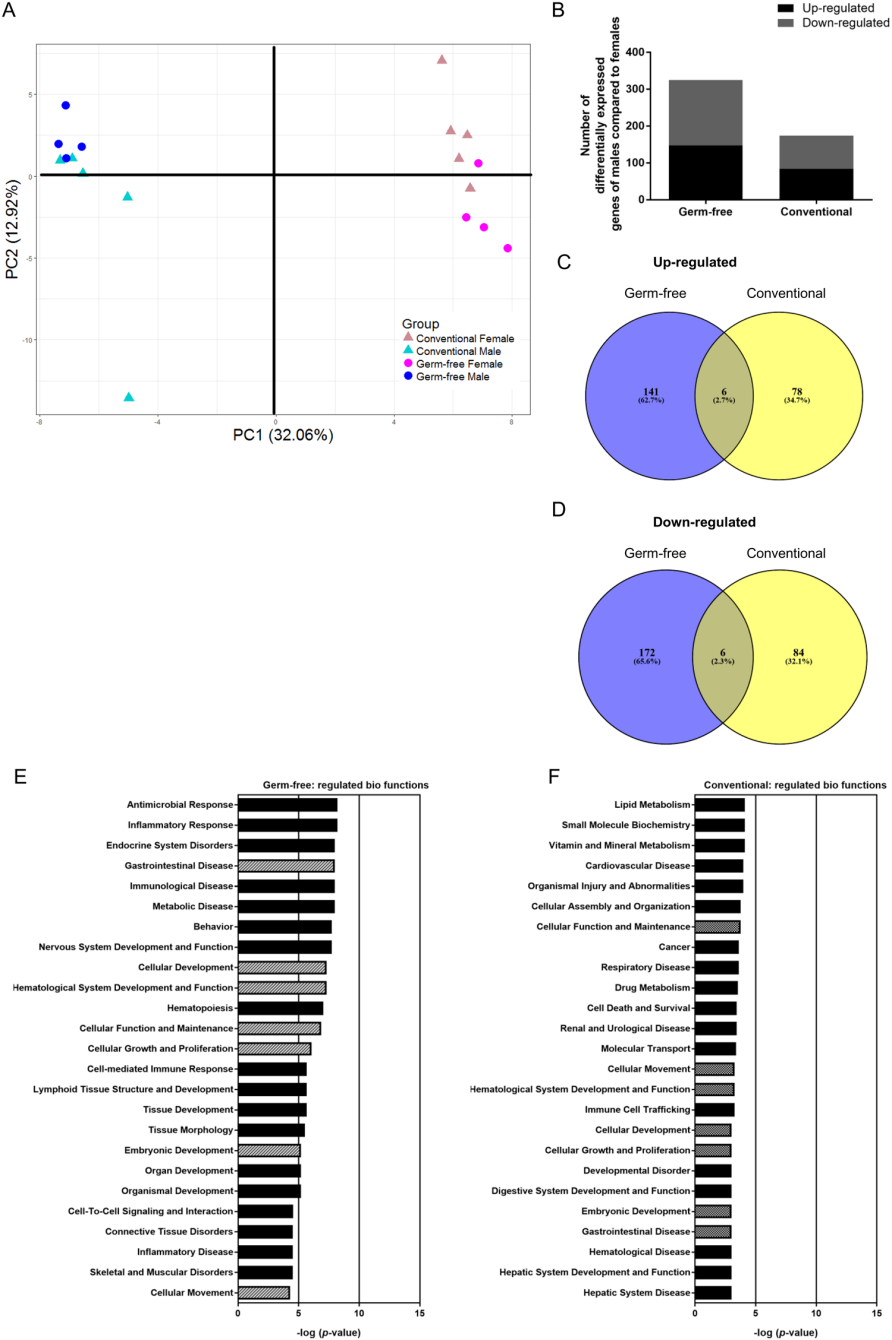
Sex-specific effects in overall ileal gene expression and related biological functions. (**A**) PCA was performed on IQR-based top 1,000 most variable genes in microarray analysis of male and female GF and Conv mice. (**B**) Significantly up- and down-regulated genes of males compared to females shown for GF and Conv mice. (**C**,**D**) Significantly up- and down-regulated genes of male compared to female (p ≤ 0.01) mice were compared between GF and Conv mice and are presented as Venn diagrams (based on intensity-based moderate t-statistics (IBMT) regularised t-test p ≤ 0.01). (**E**,**F**) IPA analysis on genes that were significantly differentially expressed between sexes in GF and Conv mice (p ≤ 0.01). The top 25 regulated biological functions in GF and Conv mice based on male-female differences are shown. Hatched bars = biological functions that occur in the top 25 of both GF and Conv mice. Filled bars = biological functions that are specific for either GF or Conv mice.

To determine to which extent sex-specific differences in lipid metabolism are influenced by presence of the gut microbiota, we performed microarray analysis in distal ileum of GF and Conv mice. Principal component analysis (PCA) of the top 1000 most variable genes, based on interquartile range of expression values across all samples, showed a distinction into two separate clusters along principal component (PC) 1, which accounts for 32.06% of the variation between samples (Fig. 1A). One cluster comprises all male samples and the other cluster all female samples. Along PC2, the samples cluster moderately (12.92%) into groups of GF and Conv mice. In total 487 genes were significantly different (p ≤ 0.01) between males and females of GF and Conv mice (Fig. 1B). From these, 147 and 84 genes were up-regulated respectively in GF and Conv mice (Fig. 1C), while 178 and 90 genes were down-regulated in GF and Conv mice, respectively (Fig. 1D). Only 6 genes, accounting for approximately 2, 5% of up- and down-regulated genes, were overlapping between GF and Conv mice, indicating a role for presence of microbiota as driver for sex differences in gene expression. Overall, from the 487 genes, there are about 1/3 more genes differentially expressed in GF than in Conv mice. This suggests that presence of microbiota reduced sex-specific gene expression. Gene expression data of the 487 differentially regulated genes between males and females were further analyzed by means of Ingenuity Pathway Analysis (IPA) to obtain insight into the differentially modulated biological functions and pathways. Based on the significance of regulation of the biological functions (−log (p-values), there are more significant sex-effects in GF mice (max −log (p-value) = 8.202) compared to Conv mice (max −log (p-value) = 4.097). Seven of the top 25 differentially regulated biological functions were overlapping in GF and Conv mice (Fig. 1E,F). These included gene sets related to gastrointestinal disease, cellular development, hematological system development and function, cellular function, cellular growth and proliferation, embryonic development and cellular movement. The top regulated biological functions specific for GF mice were related to antimicrobial and inflammatory responses most likely driven by the difference between GF male and female mice in regulation of the interferon pathway (see Supplementary Tables S3 and S4 for additional information). In contrast, in Conv mice, the top gene sets were linked to lipid metabolism and small molecule biochemistry.

### Strong sex-specific differences in sub-functions of lipid metabolism in presence of gut microbiota

**Figure 2:**
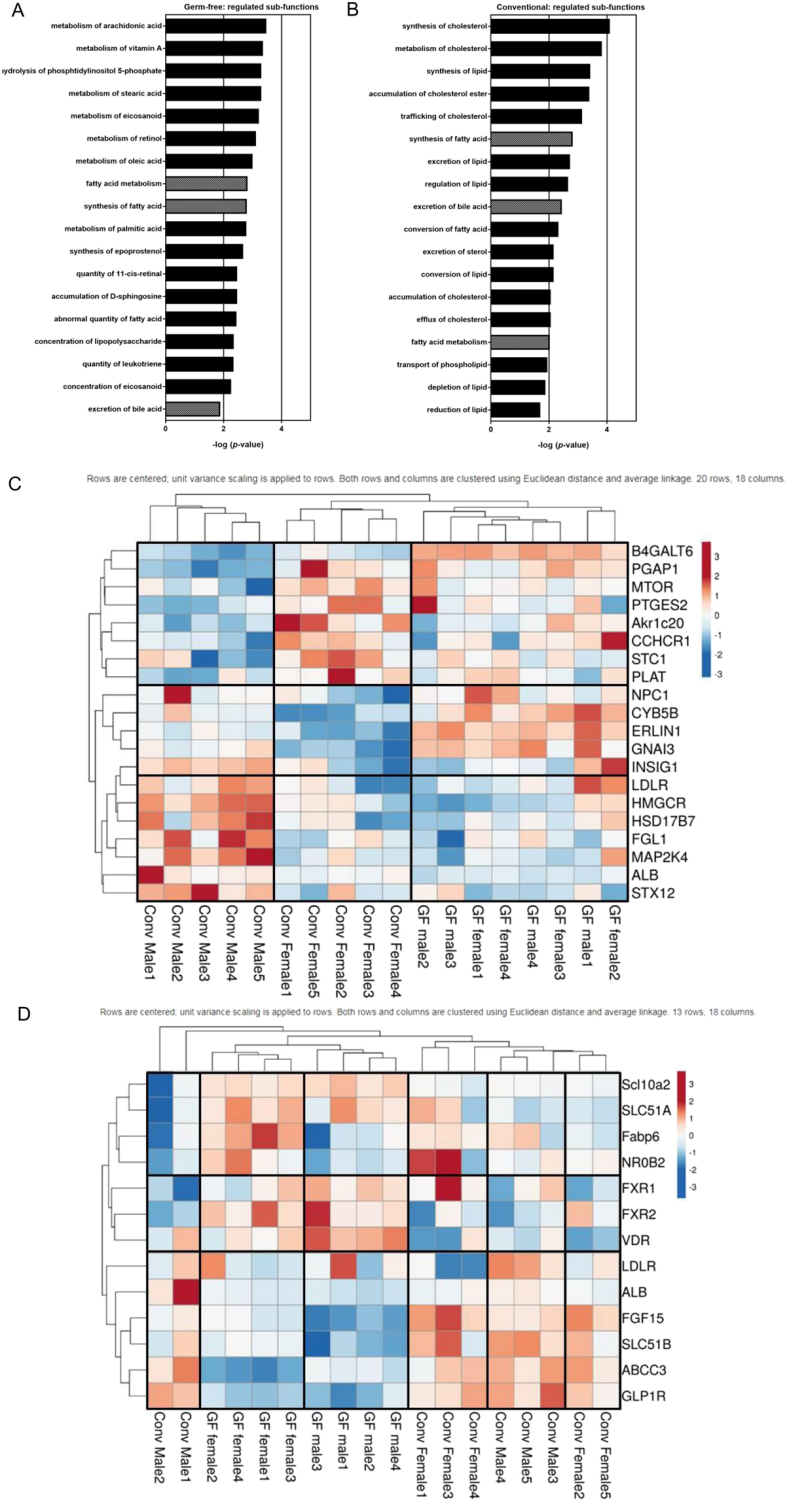
Sex-specific transcriptional effects in lipid metabolism sub-functions. (**A**,**B**) Top 18 sex-specific significantly regulated (p ≤ 0.01) sub-functions of lipid-metabolism in GF and Conv mice based on IPA. Hatched bars = sub-functions that are regulated in both GF and Conv mice. Filled bars = sub-functions that are specific for either Conv or GF mice. (**C**) Heat maps based on genes from 40 sub-functions in lipid metabolism for male versus female differences in Conv mice based on IPA. Gene expression is shown on a color scale for GF and Conv mice with blue indicating lower levels than average expression of respective group of mice; red shows higher values than average expression of respective group of mice. (**D**) Heat maps of target genes of ileum bile acid metabolism (based on KEGG database, genome net). Gene expression is shown on a color scale for Conv and GF mice. Blue shows lower levels than average expression of respective group of mice; red shows higher values than average expression of respective group of mice.

The outcomes of the top regulated biological functions resulted in examination of sub-functions lipid metabolism to gain more insight in the sex-specific regulation of lipid metabolism in GF and Conv mice. The biological function lipid metabolism was ranked as top 29 in GF mice, whereas it was ranked as number 1 in Conv mice (Fig. 1F). Exploration of sub-functions of lipid metabolism indicated that 34 sub-functions were differentially regulated in GF mice, compared to 40 sub-functions in Conv mice (p ≤ 0.01; Tables S1 and S2). In GF mice, top pathways within the sub-functions of lipid metabolism were related to very specific pathways of fatty acid metabolism while in Conv mice top pathways were related to the metabolism of cholesterol, lipid and fatty acids (Fig. 2A,B). The expression of all genes contributing to the 40 sub-functions lipid metabolism for male versus female differences in Conv mice are shown as a combined heat map for Conv and GF mice (Fig. 2C). Following hierarchical clustering, two main clusters appear to be made up of predominantly higher or lower relative expression of genes in Conv females and the opposed pattern in Conv males. This clear sex-specific clustering of genes related to lipid metabolism observed in Conv male and female mice was less pronounced in GF mice. Furthermore, the expression of these genes showed besides sex and colonization specific effects, also interaction effects for sex and microbial colonization (see Supplementary Table S5 for additional information). Bile acid metabolism is strongly associated with lipid metabolism; hence, we also explored gene expression of genes related to bile acid metabolism based on KEGG pathways by heat maps (Fig. 2D). We found a separation in GF mice between male and female and this was less apparent in Conv mice. Both in Conv and GF mice, the expression of Fgf15, important in the feedback loop controlling bile acid synthesis, showed relatively higher expression in females compared to males (p = 0.00582; fold change (FC) 3.318 in Conv mice; p = 0.00144; FC 4.822 in GF animals). Moreover, genes of bile acid metabolism showed specific sex-dependent effects in GF and Conv mice. In GF mice, the expression of Abcc3 (bile acid transporter) was relatively higher in males compared to females (p = 0.00273; FC 1.427), while the expression of Fabp6 (bile acid-binding protein) (p = 0.02980; FC −1.125) and Slc51b (intestinal basolateral bile acid transporter) (p = 0.03136; FC −1.129) was relatively lower. In Conv mice, transcription of the LDL receptor (Ldlr; p = 0.00772; FC 1.176) was relatively higher in males compared to females. Besides sex and colonization specific effects, the bile acid genes also demonstrated interaction effects (see Supplementary Table S6 for additional information).

### Presence of gut microbiota leads to differential sex-specific effects in serum bile acid composition and levels

**Figure 3:**
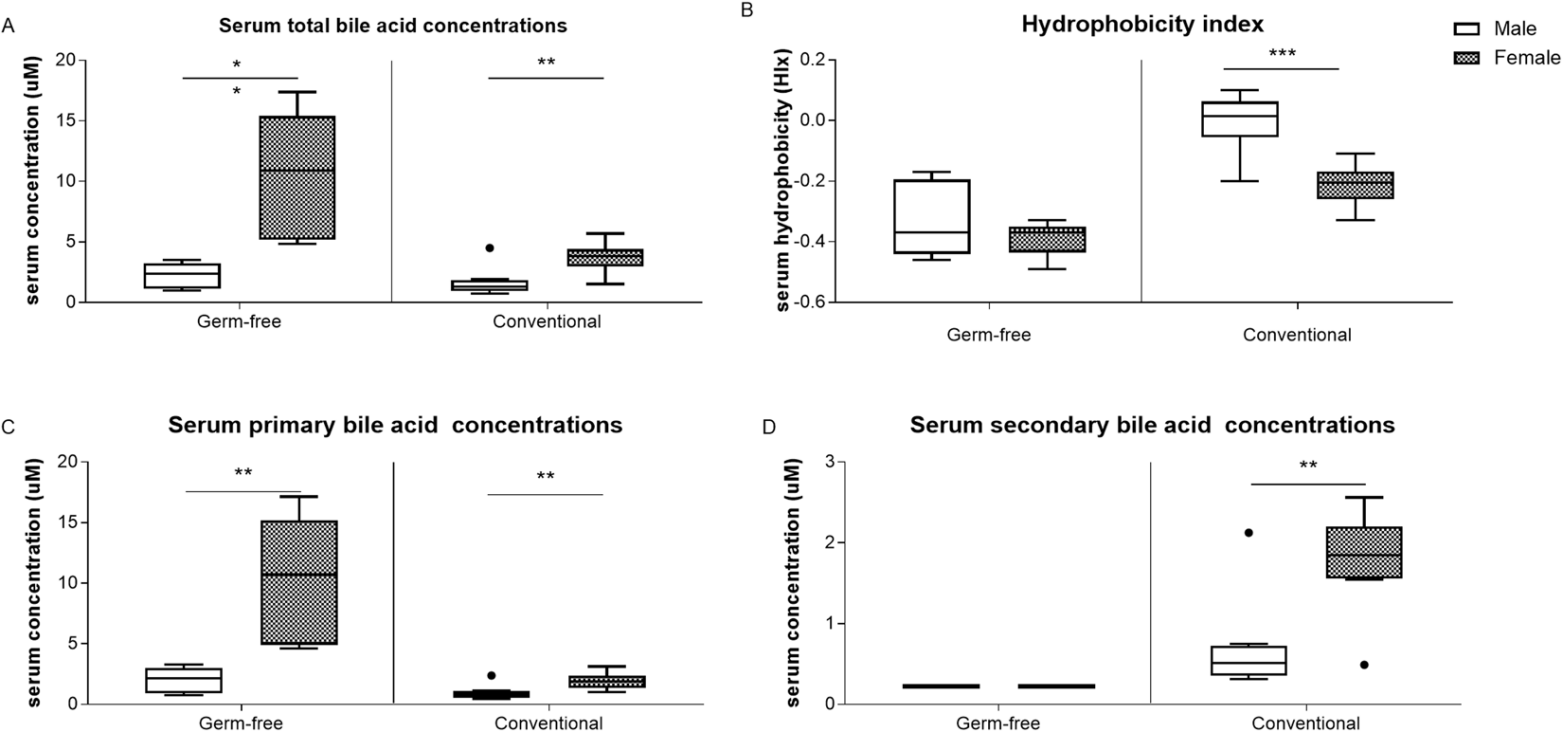
Serum bile acid concentrations. (**A**) Serum total bile acid concentrations. (**B**) Hydrophobicity index. (**C**) Serum primary bile acid concentrations. (**D**) Serum secondary bile acid concentrations. Data is presented as median and the 25–75th percentile of n ≤ 5 (GF), n ≤ 8–10 (Conv). P-values p < 0.05 were considered statistically significant and depicted as *p < 0.05; **p < 0.01 and ***p < 0.001.

**Figure 4:**
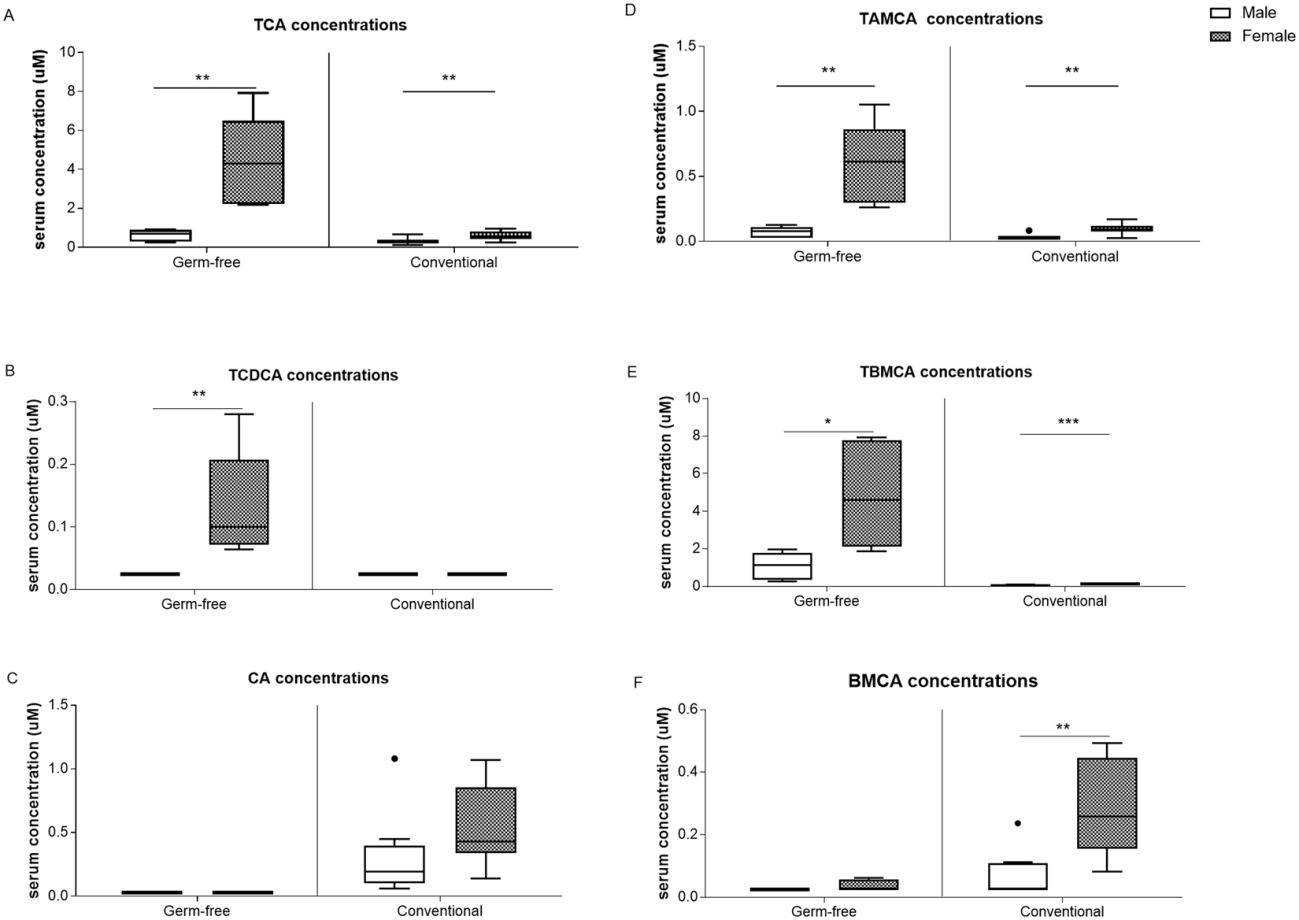
Serum primary bile acid concentrations. (**A**) TCA. (**B**) TCDCA. (**C**) CA. (**D**) TAMCA. (**E**) TBMCA. (**F**) BMCA. Data is presented as median and the 25–75th percentile of n ≤ 5 (GF), n ≤ 8–10 (Conv). P-values p < 0.05 were considered statistically significant and depicted as *p < 0.05; **p < 0.01 and ***p < 0.001.

**Figure 5:**
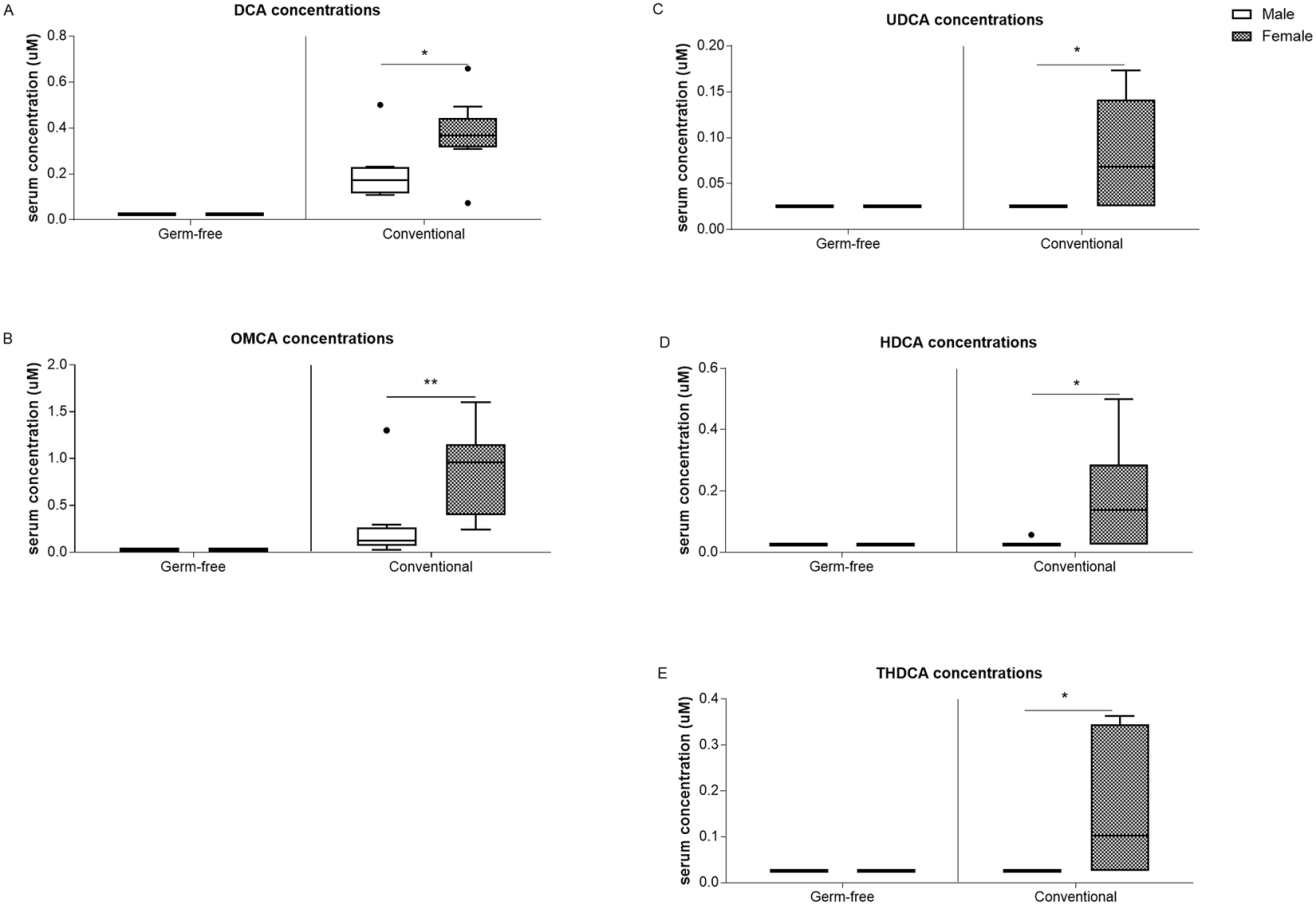
Serum secondary bile acid concentrations. (**A**) DCA. (**B**) OMCA. (**C**) UDCA. (**D**) HDCA. (**E**) THDCA. Data is presented as median and the 25th and 75th percentile of n ≤ 5 (GF), n ≤ 8–10 (Conv). P-values p < 0.05 were considered statistically significant and depicted as *<0.05; **<0.01 and ***p < 0.001.

The microarray data suggests that sex-specific differences in intestinal bile acid homeostasis is partly microbiota-driven as discussed above. To identify the contribution of gut microbiota in sex differences in bile acid homeostasis at a physiological level, bile acid profiles in serum of GF and Conv mice were measured. Serum bile acid analysis showed higher levels of total and primary bile acids in females compared to males for both GF and Conv mice (4-fold and 2-fold, respectively; Fig. 3A,C). In females, in particular serum concentrations of taurocholic acid (TCA), tauroalpha-muricholic acid (TAMCA) and taurobeta-muricholic acid (TBMCA) were higher compared to those of males (Fig. 4A,D and E). In GF females also the taurochenodeoxycholic acid (TCDCA) levels were higher compared to their male counterparts (Fig. 4B). Conv female mice had a 2.5-fold higher level of secondary bile acid species and a lower hydrophobicity index compared to Conv male mice (Fig. 3B,D). In addition, in Conv females, unconjugated and secondary bile acids beta-muricholic acid (BMCA), deoxycholic acid (DCA), omega-muricholic acid (OMCA), ursodeoxycholic acid (UDCA), hyodeoxycholic acid (HDCA) and taurohyodeoxycholic acid (THDCA) were higher compared to males (Figs 4F and 5A–E). GF mice are incapable of generating secondary bile acid species due to a lack of microbiota which is reflected in the absence of unconjugated and secondary bile acid species in serum of GF mice. The other quantified bile acid species: chenodeoxycholic acid, taurodeoxycholic acid, lithocholic acid, taurolithocholic acid, and alpha-muricholic acid did not differ between females and males (data not shown). As expected, glycine-conjugated bile acids (glycocholic acid, glycoursodeoxycholic acid, glycochenodeoxycholic acid, glycodeoxycholic acid, glycolithocholic acid and glycohyodeoxycholic acid) were below detection level.

### Sex-specific differences in microbiota composition

**Figure 6:**
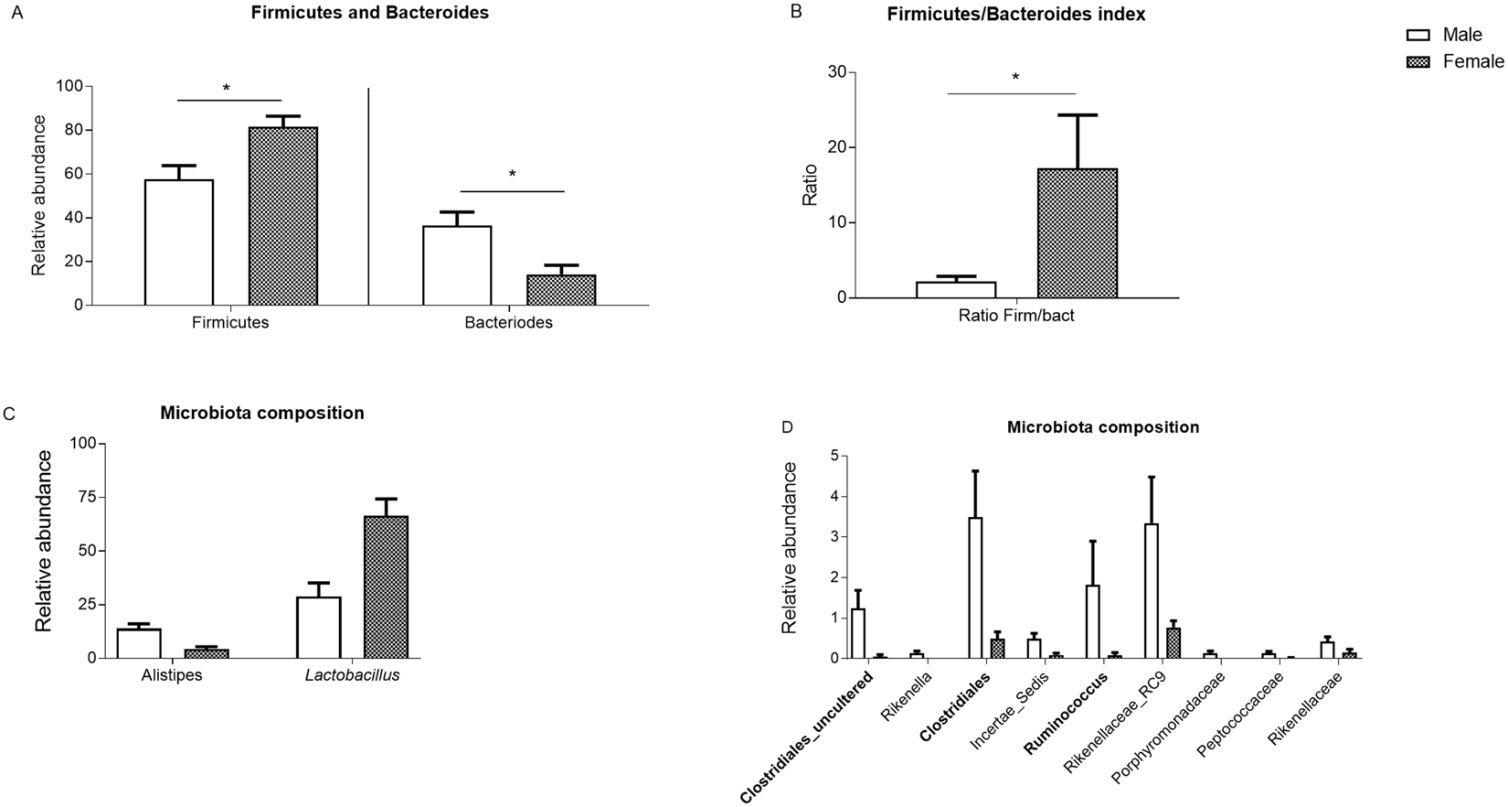
Faecal microbiota characteristics. (**A**) Firmicutes and Bacteroides of Conv mice. (**B**) Firmicutes and Bacteroides index of Conv mice. (**C**,**D**) Microbiota composition of all significant differences of the genera between males and females (p ≤ 0.05). Data is presented as the relative abundance and SEM. Bacterial BSH populations are depicted in *italics* and bacteria expressing 7α/7ß-dehydroxylase are represented in bold (based on filtered uniprot database and Ridlon 2006).

Microbiota composition was analyzed by 16S rRNA gene amplicon sequencing (Illumina Miseq) and compared between Conv males and females. The Shannon diversity index was considerably higher in males than females which is in accordance with the redundancy plot analysis (RDA; Fig. S1A,B). As shown in Fig. 6A,B, the total Firmicutes levels and Firmicutes/Bacteroides ratio index were higher in females, whereas the total Bacteroides levels were higher in males. Next, in depth microbiota composition analysis showed that there were more genera significantly higher in male mice as compared to female mice of the relevant abundant groups i.e., Alistipes and Clostridiales genera respectively (Fig. 6C,D). Further, relationships were established in co-analyzing the 3 datasets between microbial composition, ileal gene expression and serum bile acids (see Supplementary Fig. S2).

## Discussion

The present study shows that presence of the gut microbiota contributes to sex differences in lipid metabolism. Previous studies have already shown that sex and gut microbiota each regulate host metabolism, but interdependency of these two factors remained unclear^5–7,15,32,33^. The key novel finding of our study is that in the sex-specific regulation of the metabolic system the interaction between host and microbiota is important.

Male compared to female Conv mice displayed strongest differential expression in genes related to lipid metabolism, while GF mice demonstrated most pronounced sex differences in gene sets linked to gut health and inflammatory responses. Sex differences in lipid metabolism have previously been observed in both animal and human studies^5–7^ and these effects in rodents have been detected in the small intestine^7^ as well as in the liver^6^. Here, we show that predominantly cholesterol and lipid-related pathways in the distal ileum are differentially regulated in males versus females in presence of gut microbiota. Previous work demonstrated that lipid metabolism related genes were regulated in response to gut microbiota^32^. In addition, it is reported that energy metabolites and lipids in serum are modified by the gut microbiota, confirming the importance of the microbiome in host metabolism^15,34^. In agreement with previous published data, here, we also determined that there are sex differences in microbiota characteristics of Conv mice, such as Shannon diversity index and a higher diversity in microbiota composition in males compared to females^27,35^.

Our study suggests an interaction between host and microbiota in sex-specific regulation of the metabolic system. There are many mechanisms via which the gut microbiome can influence host metabolism. An increasing body of evidence confirms that regulation of host metabolic homeostasis, such as lipid metabolism, is modulated by gut microbiota through bile acids^16,21,25,36–39^. Our data showed that primary conjugated bile acid species differ between males and females, but this is not influenced by presence of gut microbiota. Similar to previous studies we did find that the presence of microbiota increased the diversity of bile acids, but decreased total bile acid concentrations^21,22^. Further, Conv females showed higher levels of secondary bile acid species than Conv males as reported previously^29^. In addition, future studies should investigate the consequences that sex differences in bile acids may have on lipid uptake.

The observed sex-specific effects on serum unconjugated and secondary bile acids may indicate a role for gut-bacteria which are capable of bile acid biotransformations^16,24,40^. In addition, an increased hydrophobicity reduces the toxicity of bile acids and leaves the glycine and/or taurine available for use as substrate and building blocks. However, this aspect is understudied and should be addressed in future studies.

BSH expression is widespread in Bacteroides, Clostridium, Lactobacillus, Enterococcus, Streptococcus, Listeria, and Bifidobacterium species (Uniprot filtered database and^23^). While Bacteroides, clostridia, *E.coli* and Ruminococcus species express on a large scale 7α/7ß-dehydroxylases^23^. In this study males had higher amounts of clostridia and *Ruminococcus* species compared to females, whereas females had a higher number of *Lactobacillus*. This difference in microbiota species could potentially be reflected in the capacity of the microbiota to induce bile salt transformations which may explain sex-specific changes in unconjugated and secondary bile acids. However, in this study, we did not measure actual BSH or 7α/β-dehydroxylase enzyme activities which could support our findings. Future research should aim to address this hypothesis. For now, we are not aware of experimental techniques that allow for *in-situ* enzyme activity assays to determine bile acid transformation capacity which is further complicated by the difficulty to assign function to phylogeny and that various microbes can perform similar functions.

Based on the heat map, sex-specific Fxr expression did not show any difference between males and females in either GF or Conv mice. However, expression of the downstream target of Fxr, Fgf15, was increased in females compared to males in both GF and Conv mice. Primary bile acids are strong activators of FXR. The sex-differences in Fgf15 expression could therefore be explained by the increased primary bile acid concentrations found in females of both GF and Conv mice. Similar findings were previously observed by Sheng and colleagues, were dietary studies in both WT and Fxr KO mice indicated an important role for Fxr activity in regulating host metabolic responses as well as driving microbiota profiles^33^. Further evidence for the reciprocal interaction between Fxr activity and microbiota comes from the finding that changes in microbiota BSH-activity correlated with increased expression of key regulators of lipid metabolism, such as Fxr, in both liver and small intestine leading to profound changes in host metabolic processes^25^.

Moreover, many genes regulating intestinal bile acid metabolism displayed more or less sex-specific expression patterns, but appeared to be influenced by the colonization status as this was less apparent in Conv mice compared to GF mice.

Together, these molecular and physiological observations provide leads in sex-specific regulation of bile acid homeostasis. Our data suggests that bile acids possibly play a role in the crosstalk between the microbiome and sex-specific regulation of lipid metabolism. We did not find an extremely strong interaction in sex-specific regulation between bile acids and microbiota activity, but the data together are compatible with a potential microbiome effect. Therefore, we hypothesize that the sex-specific effects in both primary and secondary bile acids may be influenced by an interaction between host and microbiota. We speculate that bile acid biotransformations, such as bacterial BSH activity, are different between males and females, thereby modulating sex-specific changes in unconjugated and secondary bile acids. Therefore, we hypothesize that differences in bile acid composition, could mediate sex-specific effects of host and gut microbiota on lipid regulation, thus influencing host processes such as weight gain and adiposity. Targeting the gut microbiota can be feasible in prevention or treatment of cardiovascular and other metabolic diseases. These data suggest that it is important to take a personalized and sex-specific intervention into account for the development of possible treatment strategies.

In conclusion, our data shows that presence of the gut microbiota contributes to sex differences in lipid metabolism. Further research is necessary to provide conclusive evidence in the sex-specific cross-talk regulation between microbiome and host lipid metabolism mediated by bile acids and other possible mechanisms. Overall, the current work provides a base for future research to investigate the underlying mechanisms modulated by sex and gut microbiota.

## Material and Method

### Animals

All experimental procedures applied in this study were conducted in accordance with principles of good laboratory animal care, compliant to national legislation following the EU-Directive for the protection of animals used for scientific purposes, and were approved by an independent ethics committee for animal experimentation (DEC consult, Groningen, The Netherlands). Germ-free (GF) C57BL/6JOlaHsd male and female mice (n = 5) were obtained from a breeding colony at Radboud University Nijmegen Medical Centre. Conv C57Bl/6jRccHsd male (n = 8) and female (n = 10) mice were purchased from Envigo. Conv mice were 7–10 weeks old at time of arrival and this was followed by an acclimatization period of 4 weeks to stabilize microbiota composition in these mice. All animals were housed at the facilities of Radboud University Nijmegen Medical Centre (Nijmegen, The Netherlands in a 12 h light–12 h dark cycle (light on 07.00 hours = Zeitgeber time 0 h). GF mice were housed (2–3 mice per cage) in GF isolators (Bell isolation type B30, makrolon type II, Techniplast 1284 L, Tecnilab-BMI) and Conv mice were housed (5 mice per cage) in IVC cages (Blue line polysulfon, Techniplast 1284 L, Tecnilab-BMI) with cage enrichment (Mouse igloo and Sizzle-nest). Food and autoclaved water were available ad libitum throughout the experiment. Room temperature and humidity were controlled (±22 °C and ±55%, respectively).

All mice received autoclaved rat/mouse maintenance V153X R/M-H diet (Ssniff, Soest, Germany). GF received this diet after weaning (3 weeks of age) and Conv mice after arrival (7–10 weeks of age). Samples from GF isolators were regularly screened for sterility. At the end of the experiment mice were first anesthetized with 3–5% isoflurane, bled, and sacrificed by cervical dislocation. Serum was collected and stored at −80°C. A part of terminal ileum was snap frozen in liquid nitrogen, stored at −80°C and used for RNA isolation. At time of sacrifice, GF mice were 13–15 weeks and Conv animals were 15–18 weeks old. Unfortunately, we did not have enough mice of the same age for the whole experiment due to logistical limitations.

### Serum analyses

For serum bile acid analysis 25 µL serum was used and to each sample 250 µL internal standard solution was added, vortexed for 60 s, centrifuged at 15800 × g and the supernatant poured into a clean glass tube. After evaporation under nitrogen at 40 °C the samples were reconstituted in 200 µL 50% methanol in water, vortexed for 60 s and centrifuged for 3 min at 1800 × g. The supernatant was transferred into a 0.2 µm spin-filter and centrifuged at 2000 × g for 10 min. After filtering, 10 µL of samples was injected on an Acquity UPLC BEH Column (1.7 µm × 2.1 × 100 mm) containing an Acquity UPLC BEH C18 VanGuard Pre-Column (1.7 µm × 2.1 × 5 mm) (Waters, Milford, MA, USA). The quantitative determination of bile acids was performed using a Nexera X2 Ultra High Performance Liquid Chromatography system (SHIMADZU, Kyoto, Japan), coupled to a SCIEX QTRAP 4500 MD triple quadrupole mass spectrometer (SCIEX, Framingham, MA, USA) (UHPLC-MS/MS). The LC-MS/MS system was controlled by Analyst MD 1.6.2 software. Concentrations of bile acid species measured below the linear range of 0.05 µM were indicated as 0.025 µM.

### RNA isolation

Total RNA was isolated from ileum samples using TRIzol reagent (Qiagen, Valencia, CA, USA) all according to the manufacturers’ instructions. RNA concentrations were measured using NanoDrop ND-1000 UV-vis spectrophotometer (NanoDrop Technologies, Thermo Fisher Scientific, Breda, the Netherlands). RNA quality was checked on an Agilent 2100 Bioanalyzer according to manufacturer’s instructions (Agilent, Santa Clara, CA, USA). Samples were used demonstrating intact bands of 18S and 28S ribosomal RNA subunits, showing no chromosomal peaks or RNA degradation products, and having a RNA integrity number (RIN) above 8.0.

### Microarray hybridization and analysis

100 ng of purified RNA ileum sample was labelled using the Ambion Whole Transcript (WT) Expression kit (Life Technologies Ltd, Bleiswijk, the Netherlands) and Affymetrix GeneChip WT Terminal Labelling kit (Affymetrix, Santa Clara, USA). All samples were hybridized to two 24-sample Affymetrix GeneChip Mouse Gene 1.1 ST array plates according to standard Affymetrix protocols.

An Affymetrix GeneTitan Instrument was used for hybridization, washing, and scanning of array plates. Quality control and normalisation were performed using Bioconductor software packages integrated in an on-line pipeline (https://www.ncbi.nlm.nih.gov/pubmed/21778530).

Probe sets were redefined compliant with Dai *et al*. and allocated to Entrez Gene IDs using remapped CDF (v19)^41^. Multi-array analysis pre-processing algorithm available in the Bioconductor library affyPLM was applied to acquire normalized expression estimates from raw intensity values^42^. Bioinformatic micro-array analysis, including statistics, was performed as described in Mischke *et al*.^6^. Briefly, the 21.115 defined genes were filtered for further analysis using following criteria: intensity ≥20 on at least 3 arrays an interquartile range (IQR) ≥0.2 and at least seven probes per gene. For principle component analysis (PCA), we selected the top 1000 most variable genes based on their IQR. Ingenuity pathway analysis (IPA) was applied on significantly differentially expressed genes between sexes in GF and Conv mice (p ≤ 0.01) to relate the gene expression data to biological function. Heat maps were generated using online web tool ClustVis and the Eucledian method was applied for clustering on gene and sample sets. In addition, gene sets of interest were analyzed by two-way ANOVA to investigate the effects of sex, colonization status and interaction using R 3.3.2. On the resulting p-values the false discovery rate was estimated by calculating q-values^43^. Array data were submitted to the Gene Expression Omnibus and are available under accession number GSE104063.

### Microbiota analysis

Colonic content was collected from Conv mice and snap frozen in liquid nitrogen and stored at −80 °C. These samples were used for 16S rRNA gene analysis for microbiota profiling with barcoded amplicons from V1-V2 region of 16S rRNA genes generated using a 2-step PCR strategy that decreases the impact of barcoded primers on outcome of microbial profiling)^44^. DNA extraction was carried out using a combination of bead-beating-plus column method and Maxwell 16 Tissue LEV Total RNA purification kit (Promega, Leiden, The Netherlands). The microbiota composition was determined using Illumina Miseq data analysis with a workflow employing the NG-tax pipeline^45^ and Quantitative Insights Into Microbial Ecology (QIIME) pipeline^46^. The NG-tax pipeline was used for barcode-primer filtering, de-multiplexing, out-picking, taxonomic classification, out tree-building and to create a biom file. The Biom file was further processed in QIIME for a- and b-diversity calculations. Using QIIME, Alpha diversity was calculated for Shannon observed species. Statistical tests were performed using R and using the Mann-Whitney U test with continuity correction. The Benjamini- Hochberg procedure was used to correct for multiple comparisons where appropriate with a FDR-adjusted p-value ≤ 0.05 considered significant^47^. Detailed description of microbiota analyses are described in Fransen *et al*.^48^.

### Statistics

Statistical analyses for physiological data were performed using IBM SPSS Statistics 19.0 (SPSS Benelux, Gorinchem, The Netherlands) and GraphPad Prism 6 (GraphPad Software, La Jolla, CA, USA). Variables were tested for Gaussian distribution and the nonparametric Mann-Whitney U-test was applied for serum bile acid data (SPSS Benelux, Gorinchem, The Netherlands). The following comparisons were performed for serum bile acid data, namely GF males versus females, or Conv males versus females. The difference in number of animals between Conv males and Conv females was due to the fact that 2 males died during the experiment of natural causes. The physiological data are described as median and the 25th and 75th percentile and p-values p < 0.05 were considered statistically significant and depicted as *p < 0.05; **p < 0.01 and ***p < 0.001.

Pearson’s correlation coefficients were calculated on the significantly different (male versus female) variables from the ileal gene expression, serum bile acids and microbial composition datasets to establish correlations between these three datasets. In addition, microbiota composition of genera that were significantly different between males and females were selected. In graphPad Prism 6, a p-value of p < 0.05 was considered statistically significant and a pearson correlation coefficient below −0.6 and higher than 0.6 was applied for visualization of the correlation network using Cytoscape (Cytoscape software 3.5.1).

## Electronic supplementary material

Supplementary information
