# Supplementary material for: Sex differences in lipid metabolism are affected by presence of the gut microbiota
Source: Sci Rep. 2018 Sep 7;8:13426. doi: 10.1038/s41598-018-31695-w (PMC6128923; doi:10.1038/s41598-018-31695-w)
Supplement: Supplementary file 1 — Supplementary information [file 41598_2018_31695_MOESM1_ESM.docx]

SUPPLEMENTS

TITLE: Sex differences in lipid metabolism are affected by presence of the gut microbiota

AUTHORS: Annemarie Baars*^1^, Annemarie Oosting^1^, Mirjam Lohuis^2^, Martijn Koehorst^2^, Sahar El Aidy^3^, Floor Hugenholtz^4,5^, Hauke Smidt^4,5^, Mona Mischke^1^, Mark V. Boekschoten^4,6^, Henkjan J. Verkade^2^, Johan Garssen^1,7^, Eline M. van der Beek^1,2^, Jan Knol^1,5^, Paul de Vos^4,8^, Jeroen van Bergenhenegouwen^1,7§^, Floris Fransen^4,8§^

**Table S1. List sub-functions lipid metabolism in GF mice**

| **Diseases or Functions Annotation** | **p-Value** | **Molecules** |
| --- | --- | --- |
| Metabolism of eicosanoid | 6,09E-04 | CAMP,CD74,CYP1A1,Cyp2c44,DBP,PTGDS,PTGES,PTGIS,S1PR1,SPHK2,SYK,TEF,VEGFA |
| Metabolism of arachidonic acid | 3,34E-04 | CYP1A1,Cyp2c44,DBP,TEF |
| Metabolism of vitamin A | 4,39E-04 | CYP1A1,DHRS9,RPE65 |
| Hydrolysis of phosphtidylinositol 5-phosphate | 5,01E-04 | MTM1,TMEM55B |
| Metabolism of stearic acid | 5,01E-04 | DBP,TEF |
| Metabolism of retinol | 7,55E-04 | CYP1A1,DHRS9,PLB1,RPE65 |
| Metabolism of oleic acid | 9,94E-04 | DBP,TEF |
| Fatty acid metabolism | 1,50E-03 | ABCC3,Atp8b5,CAMP,CD74,CYP1A1,Cyp2c44,DBP,ELOVL6,FGF19,Oxct2a/Oxct2b,PDK4,PHGDH,PTGDS,PTGES,PTGIS,S1PR1,SPHK2,SYK,TEF,TRIB3,VEGFA |
| Synthesis of fatty acid | 1,57E-03 | CAMP,CD74,ELOVL6,FGF19,PDK4,PTGDS,PTGES,PTGIS,S1PR1,SPHK2,SYK,TRIB3,VEGFA |
| Metabolism of palmitic acid | 1,64E-03 | DBP,TEF |
| Synthesis of epoprostenol | 2,10E-03 | PTGES,PTGIS,VEGFA |
| Accumulation of D-sphingosine | 3,39E-03 | PHGDH,SPHK2 |
| Quantity of 11-cis-retinal | 3,39E-03 | RPE65,VEGFA |
| Abnormal quantity of fatty acid | 3,60E-03 | AGPAT2,PTGES,PTGIS |
| Concentration of lipopolysaccharide | 4,48E-03 | CAMP,IL4R |
| Quantity of leukotriene | 4,53E-03 | CAMP,CYP1A1,PTGES |
| Concentration of eicosanoid | 5,48E-03 | ARNTL,CAMP,CYP1A1,PTGDS,PTGES,PTGIS |
| Conversion of prostaglandin h2 | 5,71E-03 | PTGES,PTGIS |
| Metabolism of linoleic acid | 5,71E-03 | DBP,TEF |
| Synthesis of eicosanoid | 7,02E-03 | CAMP,CD74,PTGDS,PTGES,PTGIS,S1PR1,SPHK2,SYK,VEGFA |
| Synthesis of alitretinoin | 7,08E-03 | CYP1A1,DHRS9 |
| Secretion of aldosterone | 7,47E-03 | CITED2,NFIL3,PER1 |
| Quantity of phosphatidylinositol-3-phosphate | 8,58E-03 | INPP4A,MTM1 |
| Synthesis of prostaglandin D2 | 9,66E-03 | PTGDS,PTGES,VEGFA |
| Abnormal quantity of prostaglandin | 1,02E-02 | PTGES,PTGIS |
| Synthesis of thromboxane B2 | 1,02E-02 | CAMP,PTGES |
| Activation of epoprostenol | 1,30E-02 | SPHK2 |
| Concentration of palmitoleic acid | 1,30E-02 | ARNTL |
| Elongation of lauroyl-coenzyme A | 1,30E-02 | ELOVL6 |
| Elongation of myristoyl-coenzyme A | 1,30E-02 | ELOVL6 |
| Elongation of palmitic acid | 1,30E-02 | ELOVL6 |
| Elongation of palmitoleoyl-coenzyme A | 1,30E-02 | ELOVL6 |
| Elongation of palmitoyl-coenzyme A | 1,30E-02 | ELOVL6 |
| Excretion of bile acid | 1,30E-02 | ABCC3 |

**Table S2. List sub-functions lipid metabolism in Conv mice**

| **Diseases or Functions Annotation** | **p-Value** | **Molecules** |
| --- | --- | --- |
| Synthesis of cholesterol | 7,99E-05 | HMGCR,HSD17B7,INSIG1,LDLR,NPC1 |
| Metabolism of cholesterol | 1,53E-04 | FGL1,HMGCR,HSD17B7,INSIG1,LDLR,NPC1 |
| Synthesis of steroid | 3,30E-04 | Akr1c20,CCHCR1,FGF19,HMGCR,HSD17B7,INSIG1,LDLR,NPC1,STC1 |
| Synthesis of lipid | 3,76E-04 | Akr1c20,ALB,B4GALT6,CCHCR1,ERLIN1,FGF19,GNAI3,HMGCR,HSD17B7,INSIG1,LDLR,MAP2K4,MTOR,NPC1,PGAP1,PTGES2,STC1 |
| Accumulation of cholesterol ester | 4,08E-04 | INSIG1,LDLR,NPC1 |
| Trafficking of cholesterol | 7,18E-04 | LDLR,NPC1 |
| Steroid metabolism | 1,30E-03 | CYP2B6,FGL1,HMGCR,HSD17B7,INSIG1,LDLR,NPC1,STC1 |
| Synthesis of fatty acid | 1,52E-03 | ALB,ERLIN1,FGF19,INSIG1,LDLR,MAP2K4,MTOR,NPC1,PTGES2 |
| Excretion of lipid | 1,87E-03 | FGF19,LDLR,NPC1 |
| Regulation of lipid | 2,20E-03 | FGF19,LDLR,PLAT |
| Metabolism of membrane lipid derivative | 2,35E-03 | B4GALT6,FGL1,GNAI3,HMGCR,HSD17B7,INSIG1,LDLR,MTOR,NPC1,PGAP1 |
| Efflux of choline-phospholipid | 2,57E-03 | NPC1,STX12 |
| Excretion of bile acid | 3,61E-03 | FGF19,LDLR |
| Conversion of fatty acid | 4,63E-03 | ALB,CYP2B6,HMGCR |
| Regulation of steroid | 4,82E-03 | LDLR,PLAT |
| Excretion of sterol | 6,93E-03 | LDLR,NPC1 |
| Conversion of lipid | 6,98E-03 | ALB,CYB5B,CYP2B6,HMGCR,LDLR |
| Conversion of 3-hydroxy-3-methylglutaryl-coenzyme A | 7,01E-03 | HMGCR |
| Conversion of 5(S)-HETE | 7,01E-03 | ALB |
| Crystallization of cholesterol | 7,01E-03 | ALB |
| Depletion of lactosylceramide | 7,01E-03 | B4GALT6 |
| Quantity of 1,2-dipalmitoylphosphatidylcholine | 7,01E-03 | INSIG1 |
| Reduction of 3-hydroxy-3-methylglutaryl-coenzyme A | 7,01E-03 | HMGCR |
| Accumulation of cholesterol | 8,61E-03 | INSIG1,LDLR,NPC1 |
| Efflux of cholesterol | 8,77E-03 | ALB,LDLR,NPC1,STX12 |
| Fatty acid metabolism | 9,29E-03 | ALB,B4GALT6,ERLIN1,FGF19,GNAI3,INSIG1,LDLR,MAP2K4,MTOR,NPC1,PTGES2,STX12 |
| Transport of phospholipid | 1,16E-02 | LDLR,NPC1,STX12 |
| Depletion of lipid | 1,32E-02 | B4GALT6,LDLR |
| Accumulation of farnesyl pyrophosphate | 1,40E-02 | HMGCR |
| Conversion of 12(S)-hydroxyeicosatetraenoic acid | 1,40E-02 | ALB |
| Conversion of bile acid | 1,40E-02 | LDLR |
| Quantity of alpha-tocopherol phosphate | 1,40E-02 | LDLR |
| Quantity of asialo GM2 ganglioside | 1,40E-02 | NPC1 |
| Reduction of beta-estradiol | 1,40E-02 | HSD17B7 |
| Sequestration of cholesterol | 1,40E-02 | NPC1 |
| Upregulation of corticosterone | 1,40E-02 | PLAT |
| Concentration of bile acid | 1,42E-02 | ALB,FGF19 |
| Conversion of eicosanoid | 1,42E-02 | ALB,CYP2B6 |
| Transport of long chain fatty acid | 1,42E-02 | ALB,NPC1 |
| Reduction of lipid | 1,98E-02 | HMGCR,HSD17B7 |

Table S3. List of genes that are part of top 1 regulated biological function in GF mice based on male-female differences.

| **Diseases or Functions Annotation** | **p-value** | **Molecules** |
| --- | --- | --- |
| Antimicrobial Response | 6,28E-09-1 | Oasl2,B2M,TRIM56,HLA-A,Mx1/Mx2,IFIT1B,CD8A,Fv1,DDX3X,PPM1D,IFIH1,Cd209b,PDK4,IFIT3,SNRPB,OAS2,H2-K2/H2-Q9,ZC3HAV1,OAS3,SIN3A,CAMP,EXOSC4,DDX60,SYK,STAT2 |

Table S4. List of top nine most significantly differently expressed upstream regulators in GF mice

| **Upstream Regulator** | **Expr Log Ratio** | **Molecule Type** | **Predicted Activation State** | **Activation z-score** |
| --- | --- | --- | --- | --- |
| Ifnar^*^ |  | group | Inhibited | -4,179 |
| IRF7 | -1,293 | transcription regulator | Inhibited | -4,604 |
| IFNAR1 | -1,040 | transmembrane receptor | Inhibited | -2,398 |
| TRIM24 | 1,025 | transcription regulator | Activated | 3,582 |
| PRL | 1,061 | cytokine | Inhibited | -4,022 |
| IRF3 | -1,066 | transcription regulator | Inhibited | -4,045 |
| IFNA2 |  | cytokine | Inhibited | -4,152 |
| IFNL1 |  | cytokine | Inhibited | -3,464 |
| DNASE2 | 1,451 | enzyme |  |  |

**upstream regulator Ifnar; Target molecules in dataset B2M, CD74, CD8A,HLA-A,IDO1,IFI35,IFIH1,IFIT1B,IFIT3,IRF9,Mx1/Mx2,OAS2,RNF213,Sp100,STAT2,UBE2L6,USP18,XAF1*

Table S5. False discovery rate estimations of the two-way ANOVA on the genes that were identified by IPA that contributed to the 40 sub-functions lipid metabolism for male versus female difference in Conv mice. The genes with a p-value < 0.05 for sex, colonization status, or interaction are shown.

| **Gene** | **sex q value** | **colonization status q value** | **interaction q value** |
| --- | --- | --- | --- |
| Pgap1 | 0.0196 | 0.0300 | 0.0091 |
| Insig1 | 0.0507 | 0.3116 | 0.0113 |
| B4galt6 | 0.0591 | 0.0000 | 0.0091 |
| Cyb5b | 0.0399 | 0.0005 | 0.0113 |
| Plat | 0.0125 | 0.3723 | 0.0578 |
| Hmgcr | 0.0230 | 0.0034 | 0.0113 |
| Mtor | 0.0299 | 0.2907 | 0.0091 |
| Alb | 0.0668 | 0.1292 | 0.0464 |
| Stx12 | 0.0014 | 0.0157 | 0.0546 |
| Erlin1 | 0.0014 | 0.0000 | 0.0795 |
| Akr1c20 | 0.0038 | 0.2554 | 0.0114 |
| Hsd17b7 | 0.0399 | 0.1341 | 0.0115 |
| Ptges2 | 0.0682 | 0.2739 | 0.0091 |
| Stc1 | 0.0169 | 0.2739 | 0.0367 |
| Gnai3 | 0.0007 | 0.0000 | 0.0464 |
| Ldlr | 0.0262 | 0.4022 | 0.0464 |
| Npc1 | 0.1535 | 0.1983 | 0.0094 |
| Map2k4 | 0.0426 | 0.0175 | 0.0091 |
| Fgl1 | 0.0426 | 0.0322 | 0.0091 |
| Cchcr1 | 0.0191 | 0.3199 | 0.0464 |

Table S6. False discovery rate estimations of the two-way ANOVA on target genes of ileum bile acid metabolism based on KEGG database. The genes with a p-value < 0.05 for sex, colonization status, or interaction are shown.

| **Gene** | **sex q value** | **colonization status q value** | **interaction q value** |
| --- | --- | --- | --- |
| Fgf15 | 0.0125 | 0.0037 | 0.0188 |
| Glp1r | 0.0507 | 0.0000 | 0.0300 |
| Vdr | 0.0111 | 0.0015 | 0.0831 |
| Slc10a2 | 0.1787 | 0.0015 | 0.0464 |
| Nr0b2 | 0.0138 | 0.2739 | 0.1073 |
| Fxr2 | 0.0668 | 0.0017 | 0.0578 |
| Slc51a | 0.0471 | 0.0109 | 0.0773 |
| Slc51b | 0.0507 | 0.0015 | 0.0279 |
| Abcc3 | 0.0191 | 0.0000 | 0.0513 |
| Fxr1 | 0.1674 | 0.1562 | 0.0367 |
| Fabp6 | 0.0125 | 0.2554 | 0.0115 |
| Ldlr | 0.0262 | 0.4022 | 0.0464 |

FIGURE LEGEND


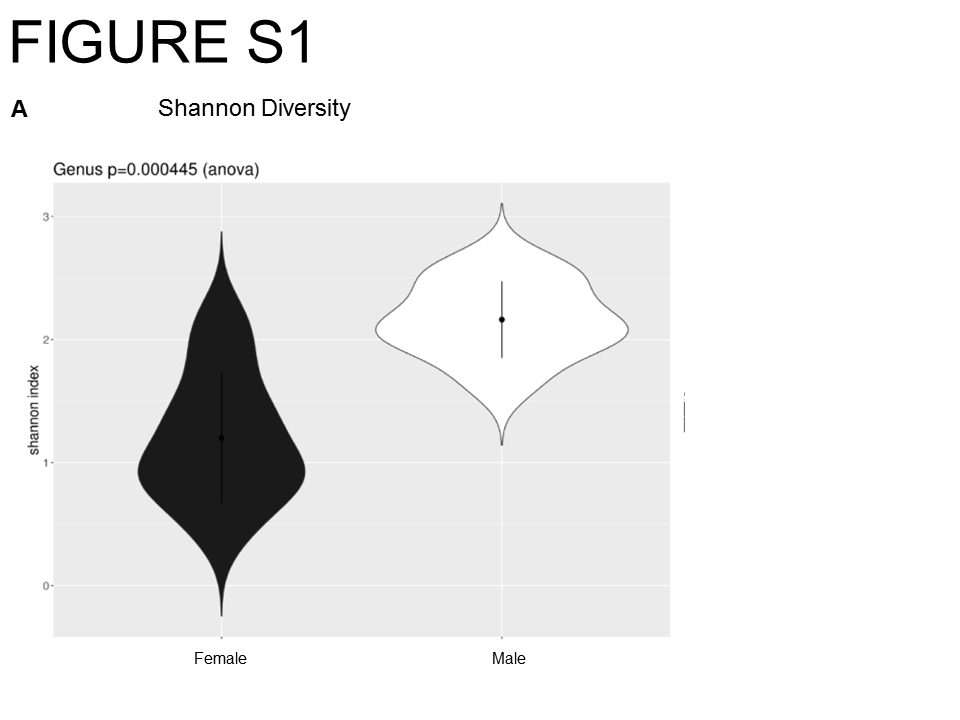


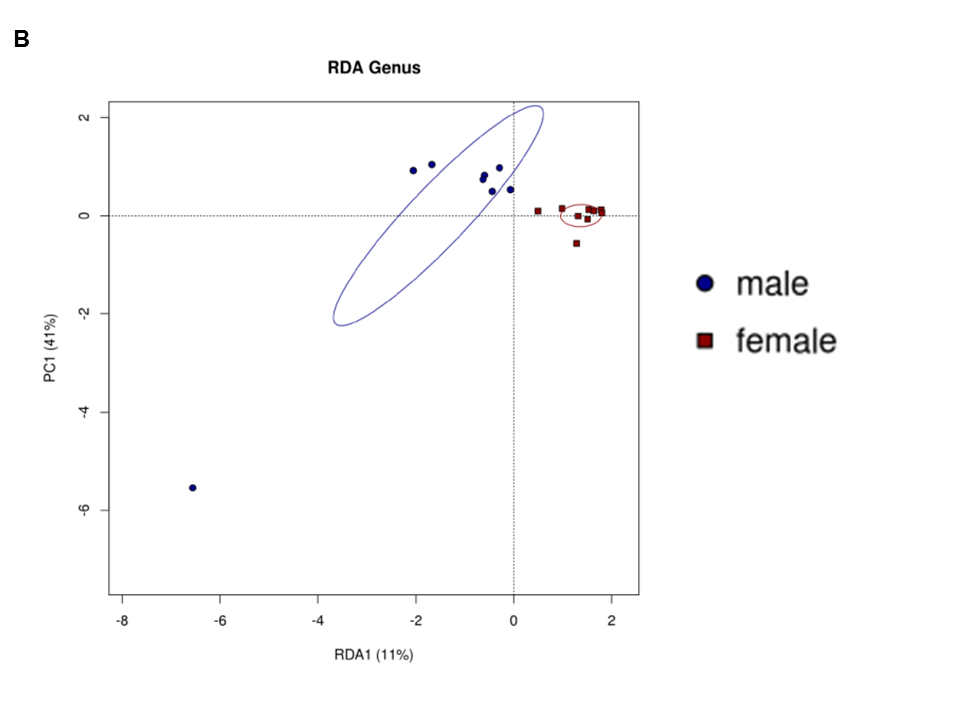


**Fig. S1: Faecal microbiota characteristics.** (A) Shannon diversity index of Conv mice. (B) RDA plot of Conv mice.

**Fig. S2: Network visualization within Cytoscape between bile acids, ileal bile acid gene expression markers and microbial composition.** Pearson's correlation coefficients were calculated on the significantly different (male versus female) variables from serum bile acids, the ileal gene expression, and microbial composition to establish correlations between these three datasets. In GraphPad prism 6, a p-value of p<0.05 was considered statistically significant and a Pearson correlation coefficient below -0.6 (shown in red as a negative correlation) and higher than 0.6 (shown in green as a positive correlation) was applied for visualization of the correlation network using Cytoscape (Cytoscape software 3.5.1). In addition, microbiota composition of genera that were significantly different between males and females are shown.

No significant correlations were detected for serum bile acids, including glycocholic acid (GCA), glycochenodeoxycholic acid (GCDCA), glycodeoxycholic acid (GDCA), Glycolithocholic acid (GLCA), glycohyodeoxycholic acid (GHDCA), alpha-muricholic acid (AMCA), glycoursodeoxycholic acid (GUDCA), lithocholic acid (LCA) and taurolithocholic acid (TLCA), for the ileal bile acid gene expression makers fatty acid binding protein (Fapb6) and low density lipoprotein receptor (Ldlr), and for microbiota composition Ruminococcus are not visualized in the network.


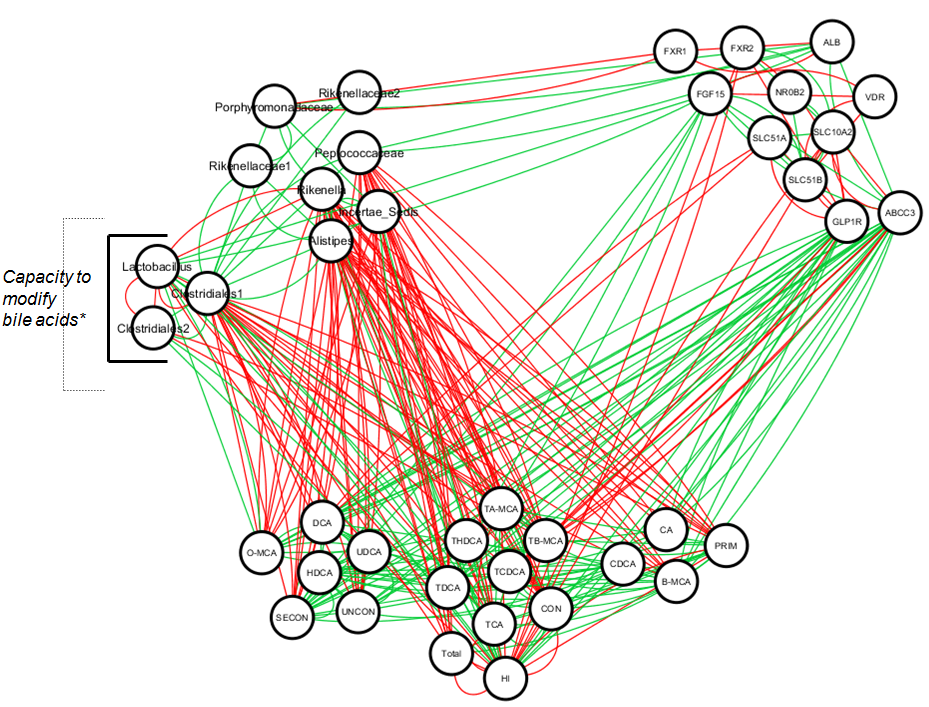


**Lactobacillus express the bile salt hydrolase gene and Clostridiales express the enzyme that is needed for 7α/β-dehydroxylation.*

*Abbreviations for serum bile acids; cholic acid (CA), taurocholic acid (TCA), deoxycholic acid (DCA), taurodeoxycholic acid (TDCA), chenodeoxycholic acid (CDCA), taurochenodeoxycholic acid (TCDCA), ursodeoxycholic acid (UDCA), tauroursodeoxycholic acid (TUDCA), beta-muricholic acid (BMCA), omega-muricholic acid (OMCA), tauro alpha-muricholic acid (TAMCA), tauro beta-muricholic acid (TBMCA), hyodeoxycholic acid (HDCA), taurohyodeoxycholic acid (THDCA), primary bile acid (PRIM), secondary bile acids (SECON), conjugated bile acids (CON), unconjugated bile acids (UNCON), total bile acids (Total) and hydrophobicity index (HI).
Abbreviations for intestinal bile acid markers; farnesoid X receptor (Fxr), fibroblast growth factor 15 (Fgf15), Albumin (Alb), nuclear receptor subfamily 0 group B member 2 (Nr0b2), solute carrier family 51 alpha subunit (Slc51a), solute carrier family 51 beta subunit (Slc51b), solute carrier family 10 member 2 (Slc10a2), glucagon like peptide 1 receptor (Glp1r) and ATP binding cassette subfamily C member 3 (Abcc3).*
